# Exploring Sleep in Caregivers of Children with Autism Spectrum Disorder (ASD) and the Relationship to Health-Related Quality of Life (HRQoL) and Family Quality of Life (FQoL)

**DOI:** 10.3390/medicina59122132

**Published:** 2023-12-07

**Authors:** Maureen Russell, Carol M. Baldwin, Stuart F. Quan

**Affiliations:** 1 Institute for Human Development, Northern Arizona University, Flagstaff, AZ 86011, USA; maureen.russell@nau.edu; 2 Edson College of Nursing and Health Innovation, Arizona State University, Phoenix, AZ 85287, USA; carol.baldwin@asu.edu; 3 Division of Pulmonary, Allergy, Critical Care and Sleep Medicine, College of Medicine, University of Arizona, Tucson, AZ 85724, USA; 4 Division of Sleep and Circadian Disorders, Brigham and Women’s Hospital, Harvard Medical School, Boston, MA 02115, USA

**Keywords:** sleep, autism spectrum disorder, quality of life, family quality of life

## Abstract

*Background and Objectives:* To investigate (1) the prevalence of sleep disorder symptoms in caregivers of children with autism spectrum disorder (ASD) and (2) the relationships between caregiver sleep problems and their health-related quality of life and family quality of life. *Materials and Methods:* Descriptive cross-sectional study of caregivers (N = 62) of children aged 6 to 11 years old diagnosed with ASD and receiving care at a regional autism research and resource center. *Results:* Participants completed the Sleep Habits Questionnaire (SHQ), the Medical Outcomes Study (MOS) SF-12, and the Beach Center Family Quality of Life Scale (FQoL). Caregivers with longer sleep duration reported better mental health and better family quality of life. Caregivers who reported insomnia symptoms, non-restorative sleep, and insufficient sleep were more likely to report poorer mental health than caregivers who did not report these sleep disorder symptoms. Caregivers with obstructive sleep apnea and restless legs syndrome experienced worse physical quality of life. *Conclusions:* The physical and mental health of the primary caregiver is essential to the support of the child with ASD and to the functioning of the family. The study findings point to the importance of future research and interventions to enhance sleep health in order to improve quality of life for caregivers of children with ASD.

## 1. Introduction

Autism Spectrum Disorder (ASD) is a complex developmental disorder that has an estimated prevalence of 1 in 54 children in the United States (U.S.) [1]. It presents challenges in many areas of early child development, such as communication, socialization, learning, and adaptive behavior [2]. These challenges can potentially impact the primary caregiver and family, as well as the child with ASD. The additional difficulties presented in advocating for services and opportunities, as well as the long-term expense needed for care of their children, create higher levels of stress for caregivers of children with ASD [3,4,5]. Additionally, the caregiving demands of a child with ASD can interfere with attending to the needs of other family members and interfere with self-care [6].

Sleep disturbances in children with ASD are reported by their parents at a prevalence of 60% to 80% [7]. Parents of children with ASD tend to be hypervigilant at night due to the possibility of unsafe activity by their children with ASD that may result in nighttime behaviors such as self-injury, property damage, or escape from their homes [8]. Additionally, the added stress related to the overall care of children with ASD may promote the development of insomnia. Although several studies have explored the health-related quality of life (HRQoL) of parents/caregivers relative to the challenges presented by children with ASD [9,10,11], there are scant data pertaining to the impact of poor sleep on HRQoL in these parents/caregivers.

Family quality of life (FQoL) is a distinct and multidimensional construct in the field of disability and acknowledges the importance of the family as the primary support and decision-maker for young children with disabilities [12]. It includes multiple domains of family wellbeing and addresses the intersection of the needs of the individual with a disability and the needs of other family members [13]. Families with children who have ASD report a more profound impact on their quality of life than families of children with other disabilities (e.g., Attention Deficit Disorder and Attention Deficit Hyperactivity Disorder) or families of typically developing children [14].

This study investigated the prevalence of sleep disorder symptoms in caregivers of children with ASD. Additionally, it explored the relationships between caregiver sleep problems and their HRQoL and FQoL. We hypothesized that caregiver sleep problems are associated with lower HRQoL and lower FQoL.

## 2. Methods 

### 2.1. Participants

#### 2.1.1. Study Design

This was a cross-sectional cohort study of family caregivers of children with ASD who were participants in Southwest Autism Research and Resource Center (SARRC) clinical or research programs in Phoenix, Arizona. SARRC promotes autism research, evidence-based practices, and community outreach to support individuals with autism throughout their lifespans [15].

#### 2.1.2. Participants and Eligibility

Participants were eligible for this study if they were the primary family caregivers for a child between 6 and 11 years old with a diagnosis of ASD confirmed by the prior administration of the Autism Diagnostic Observation Schedule [16] or the Autism Diagnostic Observation Schedule, 2nd ed. [17] by research reliable raters at SARRC. Prospective participants were excluded from this study if the child with ASD had another primary diagnosis, such as cerebral palsy or Down syndrome. Participants were also excluded if they lived outside of the United States of America or did not read and write in English.

#### 2.1.3. Recruitment

The study was initially introduced through a flyer posted at the SARRC facility and through an introductory email distributed to family caregivers in the SARRC database who met inclusion criteria. Prospective family caregivers were invited by phone or email to participate in a study investigating the role of sleep in caregivers of children with ASD and caregivers’ health-related quality of life.

Power calculations indicated that a sample size of 60 participants would provide adequate power (1 − β = 0.80) to detect a large effect for multiple regression models with two predictors: sense of coherence (SOC) and caregiver sleep. A response rate of 29% to 42% was expected based upon previous mailed survey studies with similar convenience samples [11,18]. Using a predicted response rate of 36%, 159 eligible families from the SARRC database were contacted and invited to participate. Interested caregivers were given the option of completing a questionnaire packet either through a secure online link or by mail. A total of 62 caregivers of children with ASD between the ages of 6 and 11 years ultimately completed all questionnaires.

The study was approved by the Arizona State University Human Subjects Institutional Review Board (IRB ID: STUDY00000578, Approval Date: 5 February 2014 before implementation. The study was considered exempt from the need to have a signed consent form. A cover letter, sent with the questionnaire packet, explained the intent, risks, and benefits of the study as well as informing the caregiver that participation was voluntary. The letter also stated that informed consent was implied if the completed questionnaire was returned.

### 2.2. Measures 

#### 2.2.1. Demographic Questionnaire and Brief Health History

A demographic and health history questionnaire elicited the age, gender, race/ethnicity, marital status, education status, and family income of the primary caregiver. It also asked the caregivers if a healthcare provider had ever told the participant that he or she had any of the following most common health problems: arthritis, asthma, cancer, depression, diabetes, heart disease, high blood pressure, high cholesterol, or obesity/overweight. The participant was asked to write in any additional health problems that were not listed.

#### 2.2.2. Sleep Habits Questionnaire (SHQ)

The Sleep Habits Questionnaire (SHQ), developed for the Sleep Heart Health Study, was used to obtain sleep symptoms and sleep disruptors in the caregiver participants [19]. The questionnaire addressed the following seven sleep domains:

1. Snoring was ascertained by these two questions:
  - “How often do you snore?” Possible responses included “rarely—less than one night a week”, “sometimes—1 or 2 nights a week”, “frequently—3 to 5 nights a week”, “always or almost always—6 or 7 nights a week”, and “don’t know”.
  - Participants were also asked, “How loud is your snoring?” Possible responses included “I never snore”, “only slightly louder than heavy breathing”, “about as loud as mumbling or talking”, “louder than talking”, or “extremely loud—can be heard through a closed door”, and “I don’t know”.
    Snoring was considered as present if it was occurring at least “sometimes” and was as “about as loud as mumbling or talking”.
2. Breathing pauses were assessed by these questions:
  - “Are there times when you stop breathing during sleep?” Possible responses included “yes”, “no”, and “I don’t know”.
  - “Has anyone ever told you that they saw you stop breathing during your sleep?” Possible responses included “yes”, “no”, and “I don’t know”.
    Obstructive sleep apnea was considered as present if either of the aforementioned questions related to breathing pauses was answered as “yes”.

Data pertaining to the domains of non-restorative sleep, insufficient sleep, and insomnia were elicited by asking, “Please indicate how often you experience each of the following”. Responses were rated on a 5-point Likert-like scale from ‘Never’ to ‘Almost Always’.

3. Non-restorative sleep was addressed with the question, “Feel unrested during the day, no matter how many hours of sleep you had”.
4. Insufficient sleep was addressed with the question, “Not getting enough sleep”.
5. Insomnia symptoms were assessed by inquiring about whether the participant had “trouble falling asleep”, “Wake up during the night and have difficulty resuming sleep”, and “Wake up too early in the morning and are unable to resume sleep”.
6. Restless legs syndrome (RLS) was ascertained using four questions regarding leg sensations, body position when experiencing the symptoms, time of day, and alleviation of symptoms. Participants were characterized as having RLS symptoms if they answered “yes” to all four of the following questions [20]:
  - Do you often have an urge to move your legs?
  - Is this symptom worse when you are sitting or lying down?
  - Do the symptoms improve if you get up and start walking?
  - Do the symptoms occur in the evening or at night?
7. Participants also self-reported their weekday and weekend sleep duration.

Dichotomized variables were created from the non-restorative sleep, insufficient sleep, and insomnia responses collected from the SHQ. These variables were coded as “0” for participants who endorsed having symptoms “never”, “rarely (1 day a month)”, or “sometimes (2–4 days a month)” and as “1” if the participant endorsed having symptoms “often (1–3 days a week)” or “almost always (4 or more days a week)”.

#### 2.2.3. Epworth Sleepiness Scale (ESS)

The ESS was used to assess excessive daytime sleepiness (EDS) [21]. It is a validated self-completion tool that asks participants to rate the likelihood of falling asleep in several common situations. The ESS assesses sleepiness using the question, “What is the chance that you would doze off or fall asleep” followed by a list of eight situations including “riding as a passenger in a car”, “watching TV”, and others. For each situation, possible responses include four ordinal categories ranging from “no chance” (0) to “high chance” (3). The scores range from 0 to 24 with a score of >10 suggesting EDS. A score of >10 on the ESS was coded as a dichotomized variable “1” or “yes” for excessive daytime sleepiness. A score of ≤10 was coded as “0” or “no”.

#### 2.2.4. Health-Related Quality of Life (SF-12)

The Medical Outcomes Study (MOS) SF-12 is a multi-purpose short-form generic measure of health status, developed to be a much shorter, yet valid, alternative to the SF-36 for use in extensive surveys of general and specific populations as well as large longitudinal studies of health outcomes [22,23]. All SF-12 items were derived from the MOS SF-36. Physical and mental regression weights and a constant for both measures come from the general U.S. population. The calculation of the Physical Component Summary Scale (PCS) and Mental Component Summary Scale (MCS) used the algorithm provided by Ware et al. [23], and higher scores indicate a higher perceived HRQoL. Both the PCS and MCS are transformed to have a mean of 50 and a standard deviation of 10 in the general U.S. population, with higher scores indicating better physical and mental health [23].

#### 2.2.5. Beach Center Family Quality of Life Scales (FQoL)

The Beach Center FQoL Scale consists of 25 questions within five subscales: Parenting, Family Interaction, Physical/Material Wellbeing, Emotional Wellbeing, and Disability-Related Support. The participant responds to the statement, “For my family to have a good life together: How satisfied am I that …”. followed by 25 items (e.g., “My family members have some time to pursue their own interests”). Each of the 25 items is rated on a 5-point Likert scale from 1 = “very dissatisfied” to 5 = “very satisfied”. The test–retest reliability ranges from a correlation of 0.60 to 0.77 on subscales for satisfaction between time points [24].

### 2.3. Statistical Analysis 

All data were analyzed using IBM SPSS version 22 (Armonk, NY, USA). Descriptive statistics were computed for all variables. Means and standard deviations (*SD*) were calculated for continuous variables, and percentages were reported for categorical variables. Student’s *t*-test measured associations between sleep disorder symptom variables and the dependent variables HRQoL and FQoL. For symptoms related to sleep quality and insomnia, a one-way analysis of variance using a linear contrast was performed to determine whether there was an impact of increasing severity of sleep disruption and decrements in HRQoL and FQoL. Correlations were used to determine associations between health conditions and sleep duration, HRQoL, and FQoL. Partial correlations evaluated the relationship between sleep duration and quality of life (i.e., MCS, PCS, and FQoL), controlling for caregiver age, family income, and the number of caregiver health conditions.

Caregivers reported their average sleep duration during weekdays and on the weekends. Average sleep duration was calculated as

[(average weekday sleep hours × 5) + (average weekend sleep hours × two)]/7.

The Physical (PCS) and Mental Composite Scales (MCS) derived from the SF-12 were used to describe HRQoL. Pearson correlations analyzed the strength in the relationships among caregiver age, caregiver sleep duration, HRQoL, and FQoL. The level of statistical significance was established at *p* ≤ 0.05.

## 3. Results

Table 1 provides a summary of the sociodemographic characteristics of the study participants. The caregivers who participated in the survey were, on average, 40.2 years old (*SD* = 4.4), mothers (91.9%), married (93.5%) and Non-Hispanic White (79.0%). More than half (53.2%) reported their household income to be more than $100,000 and all attended at least “some college”. Either full-time or part-time employment was reported in 58.1% of the caregivers. The respondents had an average of 2.2 dependent children (*SD* = 0.9) living with them. The mean age of the children with ASD was 7.6 (*SD* = 1.5) years, and 80.6% were boys.

The prevalence of caregiver health conditions is shown in Table 2. Depression (25.8%), asthma (17.7%), and overweight/obesity (19.4%) were the most common provider-diagnosed health conditions. Caregivers experienced a mean of 1.2 (*SD* = 1.2) health conditions with a range between 0 and 5.

Table 2 also presents the prevalence of various self-reported sleep symptoms and sleep duration. The average sleep duration was 6.4 h (*SD* = 1.0) per night but was 6.2 (*SD* = 1.0) hours of sleep on weekdays and 6.7 (*SD* = 1.1) hours of sleep on weekends. Eighty-two percent of participants had a short sleep duration, defined as ≤7 h per night. Some caregivers reported as little as 4 h of sleep per night; no caregivers reported sleep durations of >8 h per night. Insufficient sleep was reported in 54.8% of the cohort. At least one insomnia symptom was present in 54.8% of the participants, with difficulty falling asleep, difficulty getting back to sleep if they wake up during the night, and the inability to return to sleep if they wake up too early in the morning noted in 32.2%, 23.2%, and 27.4%, respectively. On average, caregivers reported that their average sleep latency was 23.3 min (*SD* = 18.8, range: 0–90 min). Sleep onset problems, defined as taking ≥30 min to fall asleep at bedtime, were reported by 40.3%. Fifty percent reported non-restorative sleep and 25.8% reported excessive daytime sleepiness (ESS > 10).

The prevalence of self-reported sleep conditions is displayed in Table 2. Restless legs syndrome was endorsed by 24.2% of the cohort. However, only one participant (1.6%) had RLS diagnosed by a health care provider. Frequent snoring (two nights a week or more) was reported by 19.3% of the participants. Symptoms of obstructive sleep apnea were reported by 9.7% of the caregivers in this study, and two caregivers (3.2%) had been diagnosed with sleep apnea by a health care provider.

Means, standard deviations, and the range of scores are reported in Table 3 for the Physical Composite Scores (PCS) and the Mental Composite Scores (MCS) on the SF-12. The Cronbach’s alpha for the SF-12 in this study was 0.74, indicating acceptable internal consistency. Overall, the PCS mean score was 51.85 (*SD* = 7.58), and the MCS score was 44.95 (*SD* = 9.34). When compared to the U.S. general population of adults aged 35–45 [23], there were no significant differences between the physical health (PCS) of the caregivers in the present study and the physical health (PCS) of the U.S. general population (*M* = 52.18, *SD* = 7.70), *t*(61) = −0.34, *p* = 0.74. However, mental health scores (MCS) are significantly lower (poorer) in the present study than the scores of the U.S. general population [23] (*M* = 44.95, *SD* = 9.34) *t*(61) = −4.34, *p* < 0.001. Caregivers who reported arthritis or asthma were more likely to report lower HRQoL in their physical health, whereas caregivers who reported heart disease were more likely to report lower HRQoL in mental health. Participants with arthritis or cancer were more likely to report a poorer FQoL. There were no significant relationships between health conditions and sleep duration or insomnia symptoms.

The means, standard deviations, and range of scores for the Beach Center FQoL scale total score and five subscales are shown in Table 3, with higher scores indicating a better FQoL [24]. The Cronbach’s alpha for the five subscales of the FQoL for this study was 0.80, which indicates good internal consistency. The means of the five domains rated from highest to lowest were (1) Physical and Material Wellbeing, (2) Parenting, (3) Family Interaction, (4) Disability Support, and (5) Emotional Wellbeing. The sequence of highest to lowest domains and the total score (M = 3.92, *SD* = 0.61) is similar to prior studies using the Beach Center FQoL Scale with U.S. families who have children with ASD (*M* = 3.5, *SD* = 0.76) *t*(105) = 4.46, *p* < 0.001 [25].

Table 4 displays the associations between sleep disorder symptoms and self-reported insomnia, obstructive sleep apnea, and restless legs syndrome. Scores on the PCS of the SF-12 were significantly lower in participants who had difficulty falling asleep (M = 48.71, *SD* = 8.55 vs. M = 53.35, *SD* = 6.58, *p* = 0.02), sleep onset ≥ 30 min (M *=* 49.37, *SD* = 8.31 vs. M = 53.53, *SD* = 6.65, *p* = 0.03), obstructive sleep apnea (M = 45.89, *SD* = 10.00 vs. 52.49, *SD* 7.10, *p* = 0.042), and restless legs syndrome. (M *=* 50.85, *SD* = 8.31 vs. M = 54.99, *SD* = 3.11, *p* = 0.006). Analysis of variance showed no changes in the PCS according to severity of insomnia or sleep disruption (results not shown). Scores on the MCS were lower for combined insomnia symptoms (M = 42.50, *SD* = 8.18 vs. M = 47.91, *SD* = 9.92. *p* = 0.02), difficulty staying asleep (M = 41.37, *SD* = 8.64 vs. M = 46.65, *SD* = 9.27, *p* = 0.04), early morning awakenings (M = 40.28, *SD* = 7.05 vs. M = 46.71, *SD* = 9.55, *p* = 0.01), non-restorative sleep (M = 40.64, *SD* = 9.9 vs. M = 49.26, *SD* = 6.28, *p* ≤ 0.001), and insufficient sleep (42.08, *SD* = 8.65 vs. M = 48.72, *SD* = 9.13, *p* ≤ 0.005). As shown in Table 5 for the MCS, there was a trend for increasing severity to be associated with worse HRQoL as a function of difficulty falling asleep and early morning awakenings; this was statistically significant for non-restorative and insufficient sleep. The presence of non-restorative sleep (M = 94.79, *SD* 14.04 vs. M = 103.3, *SD* = 16.12, *p* = 0.02) and insufficient sleep (M = 95.16, *SD* = 14.01 vs. M = 104.13, *SD* = 14.49, *p* = 0.02) were associated with lower scores on the FQoL. A lower score on the FQoL for sleep durations < 6 h approached statistical significance (M = 93.58, *SD* = 10.72 vs. M = 101.28, *SD* = 15.60, *p* = 0.06). Increasing severity of non-restorative and insufficient sleep were also associated with progressive worsening of FQoL (Table 5).

Table 6 shows the bivariate correlations between the SF-12 and FQoL and sleep duration and the number of co-morbid health conditions. There was a negative correlation between the number of caregiver health conditions and the SF-12 PCS, demonstrating that caregivers who reported more health conditions reported poorer physical health (r = 0.40, *p* < 0.05). Caregiver sleep duration was correlated with the SF-12 MCS (*r* = 0.28, *p* < 0.05) and FQoL (r = 0.37, *p* < 0.05). Therefore, caregivers with longer sleep duration reported better mental health and better FQoL. Partial correlation analyses demonstrated that the relationship between sleep duration and mental health remained when controlling for caregiver age, income, and the number of health conditions. (r_partial_ = 0.30, *p* = 0.022). Similarly, controlling caregiver age, income, and the number of health conditions did not attenuate the association between sleep duration and the FQoL (r_partial_ = 0.37, *p* = 0.004). There was also a positive correlation between the SF-12 MCS and the FQoL after controlling for sleep duration, age, and health conditions (r_partial_ = 0.44, *p* < 0.001), indicating that caregivers who reported better mental health also reported better family quality of life.

## 4. Discussion

This exploratory study found high prevalence rates of self-reported sleep disorders and symptoms of sleep disorders among caregivers of children aged 6–11 years diagnosed with ASD. Furthermore, sleep disorders and their symptoms were associated with adverse impacts on the physical and mental components of the HRQoL of these caregivers as well as the FQoL of their families. These results support the hypothesis that caregiver sleep problems lead to worse health-related individual and family quality of life.

The average sleep duration for caregivers in this study was 6.4 h, less than the recommended minimum of 7 h for adults [26]. Consistent with the low average sleep duration was our finding that 54.8% of the cohort reported that they obtained insufficient sleep. Short sleep duration in large general population studies is related to increased risk of cardiovascular disease, hypertension, diabetes, depression, early mortality, and a number of other health problems [27]. Our finding that insufficient sleep was associated with worse scores on the MCS of the SF-12 agrees with these previous results and suggests that caregivers of children with autism may be at particular risk for mental health issues. In contrast, insufficient sleep was not associated with lower scores on the PCS of the SF-12. Previous studies have observed a reduction in the PCS of the SF-36 in association with shorter sleep durations [28,29]. These latter studies focused on elderly cohorts; therefore, it is possible that the younger ages of our participants mitigated any adverse impact of short sleep duration on their physical health. Future studies that include information on health habits (i.e., diet and exercise) and relevant socioeconomic (SES) factors (e.g., access to medical care, income, and education) are necessary to understand any associations. Longitudinal studies are also needed to investigate the long-term effects of short sleep duration on the physical and mental health of caregivers of children with ASD.

Insomnia symptoms were the most frequently reported sleep problems by the caregivers of children with ASD. The prevalence of any symptom of insomnia was 54.8%, which is higher than the 27.3% reported in adults in the U.S. [30]. Possible explanations for this higher rate of insomnia include greater stress and anxiety among caregivers as well as increased rates of depression and restless legs syndrome. There is a bidirectional relationship between insomnia and depression [31]; insomnia is a risk factor for depression and, conversely, one manifestation of depression is insomnia. Importantly, 26.0% of the caregivers in the present study reported a diagnosis of depression, providing support for the role of depression as an etiology for insomnia in our cohort. Additionally, the prevalence of RLS in the cohort was 24.2%, which is higher than the 3.0% rate reported in a recent multinational systematic review of 97 studies [32]. It is likely that RLS is also contributing to the high prevalence of insomnia in our cohort, inasmuch as one of the primary consequences of RLS is insomnia [33,34].

Insomnia among the caregivers in this study was strongly associated with reductions in the MCS of the SF-12. As individual symptoms became more severe, these reductions became greater. Previous studies documented that insomnia is related to poor mental health in several chronic health conditions as well as in the general population [35]. It is likely that the high prevalence of depression in our cohort is contributing to the adverse impact of insomnia on the MCS. Previously, we documented that RLS in caregivers of children with ASD is associated an increase in insomnia symptoms and a corresponding reduction in quality of life [36]. Insomnia and RLS are treatable conditions; addressing both in caregivers provides an opportunity to improve their mental health and overall HRQoL.

Although two important symptoms of obstructive sleep apnea (OSA), snoring and excessive daytime sleepiness, did not show associations with HRQoL, our definition of OSA was related to a reduction in the PCS of the SF-12. Previous studies in the general population have demonstrated that OSA adversely impacts the PCS component of the SF-36 [37,38]. Studies of quality of life using other instruments have shown negative effects from OSA [39]. In some reports, however, only severe OSA manifested as a significant reduction in the PCS. Although the severity and treatment statuses of participants with OSA are unknown, our results are consistent with these previous studies showing an adverse impact of OSA on HRQoL. It would be important, therefore, for caregivers of a child with ASD to seek evaluation and treatment for OSA if there are any suggestive symptoms; treatment for OSA has been shown to improve quality of life [40].

Caregivers who had shorter sleep durations were more likely to report poorer FQoL. Poorer FQoL was also reported by caregivers who endorsed non-restorative sleep and insufficient sleep with greater reductions in FQoL occurring in concert with increasing symptoms of poor sleep. These relationships are noteworthy; however, the underlying explanation is unknown. In previous research, positive social relationships have been associated with more satisfactory FQoL [41]. Both sleep and social support have broad influences on physical and mental health and there is an association between greater social support and more favorable sleep outcomes [42]. Family functioning can have a significant influence on the sleep habits of the primary caregiver, the child with ASD, and other family members. Although the families in this study generally had higher incomes, families who have fewer economic resources may live in tighter living quarters that create challenges in maintaining sleep environments that are quiet, dark, and have a comfortable temperature—conditions conducive to good quality sleep [43]. Bedtime practices that promote sleep, such as consistent bedtime routines, are less likely to occur in a family who lives in poverty or overcrowded housing conditions [44,45]. Short sleep duration and sleep problems that include trouble falling and staying asleep have been associated with lower socioeconomic status [46]. Future research that includes information from multiple family informants about the sleep environment and family sleep habits as well as multiple perspectives of FQoL would be valuable.

There are limitations to this research. The study consisted of a small convenience sample of caregivers of children with ASD, and these results may not necessarily be generalizable to other caregivers of children with ASD. Additionally, because of the descriptive cross-sectional nature of this study, causality should not be inferred. This study also relied on caregivers’ self-reports of their sleep disturbances. Thus, misclassification could have occurred. Inasmuch as socioeconomic status is a likely contributor to HRQoL and FQoL among caregivers, the lack of a control group of caregivers of healthy children with similar socioeconomic status is also a limitation. Furthermore, we did not ascertain the severity of ASD symptoms of the children and were not able to determine whether decrements in HRQoL and FQoL correlated with ASD severity. The consistency of our findings demonstrating the adverse impact of sleep problems on HRQoL and FQoL, however, strengthen their validity.

Despite these limitations, there are several major strengths of this study, including a confirmed diagnosis of the children with ASD through Autism Diagnostic Observation Schedules (ADOS or ADOS-2) that have strong sensitivity and specificity for diagnoses [16,17]. Importantly, this study addressed the impact of caregiver sleep on both HRQoL and family functioning. There has been increasing attention paid to interventions and policies enhancing the value of the family as long-term foundational support for individuals with ASD and their caregivers. Such interventions as cognitive behavioral therapy to improve sleep, exercise programs, and caregiver health interventions (e.g., resilience education, mindfulness, skills to manage care recipients, and coping strategies) have been shown to be effective in improving caregivers’ sleep [47]. In addition, legislation has been introduced in several state and territorial jurisdictions in the United States to provide economic support (e.g., subsidizing cost of childcare and caregiver tax credits) for caregivers as well as nurse home-visiting programs [48]. This study supports such initiatives and sheds light on the importance of caregiver sleep and health and their subsequent effect on FQoL.

## 5. Conclusions

Among caregivers of children with ASD, the prevalence of symptoms of sleep disorders and self-reported sleep disorders is high and is associated with an adverse effect on individual HRQoL and FQoL. The physical and mental health of the primary caregiver is essential to the support of the child with ASD. Our study supports employing policies providing economic support and interventions, such as cognitive behavioral therapy, exercise programs, and caregiver health interventions to improve the sleep of caregivers of children with ASD.

## Figures and Tables

**Table 1:** Sociodemographic characteristics of family caregivers (N = 62).

	n	Percentage or Mean (*SD*)
Relationship		
Mother	57	91.9%
Father	5	8.1%
Caregiver age	62	40.2 (4.4)
Race/ethnicity		
Asian	1	1.6%
Black or African American	3	4.8%
Hispanic	6	9.7%
Non-Hispanic White	49	79.0%
Pacific Islander	1	1.6%
Other	2	3.2%
Educational Level *		
Some college	17	27.4%
4-year degree	29	46.8%
Graduate degree	16	25.8%
Marital status		
Married	58	93.5%
Divorced	3	4.8%
Other	1	1.6%
Employment status		
Employed full-time	23	37.1%
Employed part-time	13	21.0%
Unemployed/retired	15	24.2%
Other	11	17.7%
Current household income		
Under $30,000	1	1.6%
$30,000 to $39,999	0	0.0%
$40,000 to $49,999	2	3.2%
$50,000 to $59,999	5	8.1%
$60,000 to $69,999	4	6.5%
$70,000 to $79,999	6	9.7%
$80,000 to $89,999	3	4.8%
$90,000 to $99,999	8	12.9%
More than $100,000	33	53.2%
Number of dependent children	62	2.2 (0.9)
Age of child with ASD	62	7.6 (1.5)
Gender		
Boy	50	80.6%
Girl	12	19.3%

* All participants were high school graduates.

**Table 2:** Caregiver health conditions and sleep disorder symptoms (N = 62).

Health Conditions	n = Yes	Percent = Yes	n = No	Percent = No
Arthritis	6	9.7%	56	90.3%
Asthma	11	17.7%	51	82.3%
Cancer	4	6.5%	58	93.5%
Depression	16	25.8%	46	74.2%
Diabetes	1	1.6%	61	98.4%
Heart Disease	1	1.6%	61	98.4%
High Blood Pressure	6	9.7%	56	90.3%
High Cholesterol	4	6.5%	58	93.5%
Overweight/obese	12	19.4%	50	80.6%
**Sleep Disorder Symptoms**				
Insomnia Symptoms	34	54.8%	28	45.2%
Difficulty falling asleep	20	32.2%	42	67.7%
Difficulty staying asleep	20	32.2%	42	67.7%
Early morning waking	17	27.4%	45	72.6%
Sleep onset ≥ 30 min	25	40.3%	37	59.6%
Non-restorative Sleep	31	50.0%	31	50.0%
Insufficient Sleep	34	54.8%	28	45.2%
Excessive Daytime Sleepiness (EDS) *	16	25.8%	46	74.2%
Snoring	12	19.3%	50	80.6%
Obstructive Sleep Apnea	6	9.7%	56	90.3%
Restless Leg Syndrome (RLS)	15	24.2%	47	75.8%
Sleep duration over 7 days		Mean 6.4 h	(*SD* 1.0)	
Sleep onset in minutes		Mean 23.3 min	(*SD* 18.8)	
Epworth Sleepiness Scale (ESS)		Mean 7.6	(*SD* 4.7)	

* Epworth Sleepiness Scale scores > 10 were coded as Excessive Daytime Sleepiness or EDS.

**Table 3:** Quality of Life Measures: Means and Standard Deviations (*SD*s) (N = 62).

Quality of Life Variables	Mean	*SD*	Range of Scores
SF-12 (PCS)	51.85	7.58	32.06–63.96
SF-12 (MCS)	44.95	9.34	22.30–60.51
Beach Center FQoL	3.92	0.61	2.10–5.00
Physical and Material Wellbeing	4.47	0.65	2.60–5.00
Parenting	4.04	0.73	1.67–5.00
Family Interaction	4.00	0.78	1.00–5.00
Disability Support	3.92	0.78	1.25–5.00
Emotional Well-being	3.29	1.02	1.00–5.00

Note. SF-12 (PCS) = Short-form Physical Composite Score; MCS = Mental Composite Score; FQoL = Family Quality of Life; *SD* = Standard Deviation. Subdomains of FQoL are listed from highest to lowest means.

**Table 4:** Comparison of HRQoL and FQoL measures to presence or absence of sleep disorders or sleep symptoms.

	HRQoL PCS				HRQoL MCS				FQoL			
Caregiver Sleep Disorder Symptoms	With *M* (*SD*)	Without *M* (*SD*)	*T* Score *p* Value	*df*	With *M* (*SD)*	Without *M* (*SD*)	*T* Score *p* Value	*df*	With *M* (*SD*)	Without *M* (*SD*)	*T* Score *p* Value	*df*
Insomnia symptoms	50.59 (8.16)	53.38 (6.63)	1.46 0.15	60	42.50 (8.18)	47.91 (9.92)	2.3 0.02 *	60	97.19 (15.57)	101.30 (13.49)	1.10 0.28	60
Difficulty falling asleep	48.71 (8.55)	53.35 (6.68)	2.33 0.02 *	60	42.25 (7.43)	46.23 (9.94)	1.59 0.12	60	98.13 (16.12)	99.49 (14.15)	0.34 0.74	60
Difficulty staying asleep	50.14 (7.26)	52.67 (7.68)	1.23 0.22	60	41.37 (8.64)	46.65 (9.27)	2.14 0.04 *	60	96.83 (17.89)	100.10 (13.02)	0.82 0.42	60
Early morning waking	51.79 (6.81)	51.88 (7.92)	0.04 0.97	60	40.28 (7.05)	46.71 (9.55)	2.52 0.01 *	41.83 ^a^	94.50 (15.72)	100.76 (14.09)	1.51 0.14	60
Sleep duration < 6 h	50.82 (8.68)	52.27 (7.15)	0.68 0.63	60	42.08 (10.11)	46.11 (8.85)	1.56 0.12	60	93.58 (10.72)	101.28 (15.60)	1.91 0.06	60
Sleep onset ≥ 30 min	49.37 (8.31)	53.53 (6.65)	2.19 0.03 *	60	43.89 (8.11)	45.66 (10.13)	0.73 0.47	60	98.60 (14.04)	99.35 (15.31)	0.20 0.85	60
Non-restorative sleep	50.65 (8.57)	53.06 (6.36)	1.26 0.21	60	40.64 (9.97)	49.26 (6.28)	4.07 <0.001 *	50.55 ^a^	94.79 (11.92)	103.30 (16.12)	2.36 0.02 *	60
Insufficient sleep	50.48 (8.81)	53.54 (5.57)	1.65 0.10	56.45 ^a^	42.08 (8.65)	48.72 (9.13)	2.90 0.005 *	60	95.16 (14.01)	104.13 (14.49)	2.45 0.02 *	60
Excessive daytime sleepiness (ESS > 10)	50.65 (7.45)	52.27 (7.67)	0.73 0.47	60	44.32 (9.01)	45.16 (9.53)	0.31 0.76	60	96.06 (17.67)	100.08 (13.58)	0.94 0.35	60
Snoring	50.25 (8.34)	52.25 (7.43)	0.84 0.40	60	49.35 (6.15)	43.89 (9.70)	−1.86 0.07	60	103.49 (13.27)	97.98 (14.95)	−1.17 0.25	60
Obstructive Sleep Apnea (OSA)	45.89 (10.00)	52.49 (7.09)	2.08 0.04 *	60	46.79 (9.14)	44.75 (9.41)	−0.51 0.62	60	99.42 (5.82)	99.01 (15.37)	−0.13 0.95	14.55 ^a^
Restless Leg Syndrome (RLS)	50.85 (8.31)	54.99 (3.11)	−2.85 0.006 *	58.30 ^a^	41.76 (9.61)	45.97 (9.11)	1.54 0.13	60	95.80 (19.46)	100.08 (12.91)	0.80 0.43	18.10 ^a^

Note. HRQoL (PCS) = Health-Related Quality of Life (Physical Composite Score); HRQoL (MCS) = Health-Related Quality of Life (Mental Composite Score); FQoL = Family Quality of Life; ^a^ The *t* and the *df* were adjusted because variances were not equal. * *p* < 0.05.

**Table 5:** Comparison of HRQoL (MCS) and FQoL measures according to severity of insomnia and sleep disruption symptoms.

SF-12 Mental Composite Score (MCS)												
	N	Never	N	Rarely (1 Days/Mon)	N	Sometimes (2–4 Days/Mon)	N	Often (1–3 Days/Week)	N	Always (≥4 Days/Week)	F	*p* Value
Trouble falling asleep	6	46.6 ± 5.5	17	47.8 ± 11.1	19	44.8 ± 10.1	14	42.0 ± 7.3	6	42.9 ± 8.4	2.547	0.116
Difficulty staying asleep	2	47.0 ± 2.9	18	47.1 ± 9.2	22	46.3 ± 9.9	12	41.2 ± 8.5	8	41.6 ± 9.4	3.504	0.066
Early morning awakening	11	48.1 ± 5.9	15	45.1 ± 10.3	19	47.2 ± 10.8	14	40.0 ± 7.5	3	41.4 ± 5.6	3.923	0.052
Non-restorative sleep	3	53.3 ± 4.4	6	47.6 ± 5.4	22	49.2 ± 6.7	16	43.4 ± 10.4	15	38.8 ± 9.4	16.145	<0.001
Insufficient sleep	4	52.0 ± 4.4	13	50.2 ± 7.1	22	43.6 ± 8.3	16	44.0 ± 11.3	7	37.8 ± 7.8	10.514	<0.001
**Beach Center FQoL Score**												
Trouble falling asleep	6	88.3 ± 16.8	17	104.8 ± 14.5	19	98.3 ± 11.0	14	97.0 ± 15.5	6	100.8 ± 18.7	0.033	0.856
Difficulty staying asleep	2	93.5 ± 10.6	18	100.0 ± 14.3	22	100.8 ± 12.5	12	98.2 ± 15.3	8	94.8 ± 22.2	0.347	0.558
Early morning awakening	11	100.5 ± 16.7	15	100.0 ± 14.3	19	101.5 ± 14.0	14	94.5 ±11.1	3	94.3 ±34.7	1.076	0.304
Non-restorative sleep	3	108.0 ± 19.3	6	108.2 ± 12.4	22	101.3 ± 16.9	16	95.5 ± 11.5	15	94.0 ± 12.7	6.436	0.014
Insufficient sleep	1	122	6	100.5 ± 6.0	20	104.3 ± 16.0	16	95.7 ± 12.4	19	94.6 ± 15.2	5.522	0.022

Note. HRQoL (MCS) = Health-Related Quality of Life (Mental Composite Score); FQoL = Family Quality of Life.

**Table 6:** Bivariate correlations of quality of life measures with caregiver health and sleep duration.

	1	2	3	4	5
1. Caregiver number of health conditions	_____	0.06	−0.17	−0.40 *	−0.01
2. Caregiver sleep duration		____	0.37 *	0.09	0.28 *
3. FQoL			_____	0.05	0.40 *
4. SF-12 (PCS)				_____	−0.22
5. SF-12 (MCS)					_____

Note. FQoL = Family Quality of Life; SF-12 (PCS) = Short-form Physical Composite Score; SF-12 (MCS) = Short-form Mental Composite Score. * *p* < 0.05.

## Data Availability

The data presented in this study are available on request from the corresponding author.
